# A New Signaling Pathway for HCV Inhibition by Estrogen: GPR30 Activation Leads to Cleavage of Occludin by MMP-9

**DOI:** 10.1371/journal.pone.0145212

**Published:** 2016-01-05

**Authors:** Laura Ulitzky, Manuel M. Lafer, Mark A. KuKuruga, Erica Silberstein, Nicoleta Cehan, Deborah R. Taylor

**Affiliations:** Center for Biologics Evaluation and Research, US Food and Drug Administration, Silver Spring, MD, 20993–0002, United States of America; Inserm, U1052, UMR 5286, FRANCE

## Abstract

Poor outcome in response to hepatitis C virus, including higher viral load, hepatocellular carcinoma and cirrhosis, is more associated with men and postmenopausal women than with premenopausal women and women receiving hormone replacement therapy, suggesting that β-estradiol plays an innate role in preventing viral infection and liver disease. Consequently, most research in the field has concluded that estrogen affects HCV replication through viral interactions with estrogen receptor-α. Previously, estrogen-like antagonists, including Tamoxifen, were shown to reduce HCV RNA production and prevent viral entry, although the authors did not identify host factors involved. Estrogen can act alternatively through the membrane-bound G-protein-coupled estrogen receptor, GPR30. Here, human hepatoma Huh7.5 cells were infected with HCV J6/JFH-1 and treated with estrogen or Tamoxifen, resulting in a marked decrease in detectable virus. The effect was mimicked by G1, a GPR30-specific agonist, and was reversed by the GPR30-specific antagonist, G15. While previous studies have demonstrated that estrogen down-regulated occludin in cervical cancer cells, its action on liver cells was unknown. Occludin is a tight junction protein and HCV receptor and here we report that activation and cellular export of MMP-9 led to the cleavage of occludin upon estrogen treatment of liver cells. This is the first report of the cleavage of an HCV receptor in response to estrogen. We also identify the occludin cleavage site in extracellular Domain D; the motif required for HCV entry and spread. This pathway gives new insight into a novel innate antiviral pathway and the suboptimal environment that estrogen provides for the proliferation of the virus. It may also explain the disparate host-virus responses to HCV demonstrated by the two sexes. Moreover, these data suggest that hormone replacement therapy may have beneficial antiviral enhancement properties for HCV-infected postmenopausal women and show promise for new antiviral treatments for both men and women.

## Introduction

Nearly 150 million people in the world are infected with Hepatitis C virus (HCV). Vaccine development has not been successful, but advances in therapy have been dramatically improved. Finding optimal therapy combinations, including those that use host-directed antiviral mechanisms, may be prudent in the event that drug-resistant strains may arise.

Regardless of etiology, HCV infection leads the two sexes to progress to liver disease unequally. Hepatocellular carcinoma and cirrhosis are more frequent in men and post-menopausal women than in premenopausal women [1]. Moreover, postmenopausal women respond to antiviral therapy as poorly as men [2], and progression of fibrosis in postmenopausal patients was lower in women who received hormone replacement therapy (HRT) compared with untreated [1] and ovariectomized women [3], suggesting that estradiol (E2) may have an anti-fibrotic or antiviral effect. Furthermore, E2 therapy resulted in reduced liver disease in a male HCV patient [4] and in mouse models [5].

Interferon-alpha (IFNα) therapy is approved for use in the treatment of chronic HCV. When comparing response rates to IFNα therapy, men showed little difference in response to IFNα therapy based on age, but premenopausal women responded 75% of the time while women over 40 years showed only a 15.6% response to IFNα therapy [6]. This suggests that E2 may be associated with a successful response to therapy and clearance of HCV [6] and that HRT may enhance the effectiveness of drug response in postmenopausal women.

The largest amount of E2 is produced before menopause by the ovaries. The classical mechanism of E2 action is through two nuclear E2 receptors (ER-α and ER-β) that stimulate gene expression by acting as transcription factors [7]. One non-classical mechanism of E2 action is through GPR30, also known as G protein-coupled estrogen receptor (GPER) [8], predominantly found in the membrane of the endoplasmic reticulum. GPR30, a seven-transmembrane steroid receptor, promotes rapid signaling events through Zn^2+^-dependent matrix metalloproteinases (MMPs), epidermal growth factor (EGFR), PI3-kinase, calcium mobilization, and nitric oxide production [7, 9, 10].

There are several selective ER modulators (SERMs) that act as both ER antagonists and agonists [11]. The ER antagonist Tamoxifen (Tam) blocked the signaling ability of the nuclear ER and inhibited HCV infection, attachment and entry [12]. As a SERM compound, Tam is a nuclear ER antagonist in some tissues, and a GPR30 agonist in others [7].

Epithelial cells have tight junctions (TJ) that form a barrier regulating cellular permeability and may function as a component of the innate immune system to prevent viral entry or superinfection. Several viruses, including HCV, utilize the TJs to gain viral entry and spread, whereby disruption of TJs decreases HCV virus transport between adjacent cells [13]. Specifically, HCV uses the TJ proteins claudin-1 and occludin to enter hepatic cells [14, 15]. Studies showed that HCV-infected cells were resistant to infection when occludin was down-regulated, most probably due to a mechanism that prevents superinfection [16].

MMPs are zinc-dependent proteases of extracellular matrix proteins that can also cleave other molecules such as TJ proteins. In cervical cancer cells, occludin protein was down-regulated by E2 through proteolytic cleavage by MMP-7, leading to tight junction destabilization [17, 18], further explaining the observation that TJs were disrupted during zinc deficiency [19]. In our study, HCV genotype 2a (J6/JFH-1)-infected Huh7.5 cells showed a marked decrease in detectable virus when treated with E2. E2, Tam and G1 (a GPR30-specific agonist) down-regulated virus production, which was reversed by G15 (a GPR30-specific antagonist). Furthermore, E2 down-regulated the tight junction proteins and HCV receptors; occludin and claudin-1. We also show that MMP inhibitors reversed the antiviral effect of E2 by inhibiting loss of functional occludin; leading to the conclusion that active MMP-9 (gelatinase/type IV collagenase) was responsible for occludin cleavage. Here, we establish a new role for E2 in the pathway that leads to cleavage of an HCV receptor, occludin, which provides protection from HCV infection and HCV-associated liver diseases.

## Materials and Methods

### Chemical reagents and antibodies

17β-estradiol, Tamoxifen, and MMP Inhibitors SB-3CT **(**2-[[(4-phenoxyphenyl)sulfonyl]methyl]-Thiirane) and CP-471474 (2-[[[4-(4-Fluorophenoxy)phenyl]sulfonyl]amino]-N-hydroxy-2-methylpropanamide), were obtained from Sigma-Aldrich. Universal type I Interferon (Human Interferon Alpha A/D) was obtained from PBL Interferon Source. GPR30 agonist, G1 (1-(4-(6-Bromobenzo[1,3]dioxol-5-yl)-3a,4,5,9b-tetrahydro-3H-cyclopenta[c]quinolin-8-yl)-ethanone), GPR30 antagonist, G15 (*cis*-4-(6-Bromo-benzo[1,3]dioxol-5-yl)-3a,4,5,9b-tetrahydro-3H-cyclopenta[c]quinoline) and MMP inhibitor, ONO-4817 (2S,4S)-N-Hydroxy-5-ethoxymethyloxy-2-methyl-4-(4-phenoxybenzoyl) aminopentanamide, and MMP408 (S)-2-(8-(Methoxycarbonylamino)dibenzo[b,d]furan-3-sulfonamido)-3-methylbutanoic acid, were obtained from Calbiochem. GM6001, known as Ilomastat, or N-[(2R)-2-(hydroxamidocarbonylmethyl)-4-methylpentanoyl]-L-tryptophan methylamide was obtained from Millipore.

Cyclin-D1 mouse monoclonal antibody, claudin-1 and MMP-9 rabbit polyclonal antibodies were obtained from Cell Signaling Technology. Rabbit polyclonal occludin antibody (Invitrogen); HCV-core mouse monoclonal antibody (Thermo Scientific); Scavenger receptor SR-B1 rabbit polyclonal antibody (Novus Biologicals); rabbit polyclonal anti-GAPDH antibody (Trevigen); Secondary horseradish peroxidase (HRP)-conjugated antibodies were obtained from Kirkegaard & Perry laboratories.

### Cells

MCF7 cell extract (Santa Cruz) was used as a positive control for cyclin-D1 antibody. Human hepatoma Huh7.5 cells were maintained in complete Dulbecco's modified Eagles Medium (DMEM) containing 10% FBS, L-Glutamine and non-essential amino acids (Invitrogen). Cells were grown in Phenol Red Free DMEM containing 10% heat-inactivated Charcoal Stripped FBS (CSFBS) (DMEM-E2minus), L-Glutamine and non-essential amino acids were used for five days before performing the experiments with Estrogen, G1 or Tamoxifen. All FBS was screened by PCR to ensure the absence of Bovine viral diarrhea virus as described previously [20]. Cells were seeded at a high density; 5x10^4^ cells/well (96 well plate) for the inhibition studies, and 2x10^5^ cells/well in 6-well plate and 2.5x10^6^ cells/ T25 flask for protein and RNA expression studies, respectively. At high densities, Huh7.5 cells grow in layers, which enhance the number of tight junctions [21] and occludin expression.

### Preparation of HCV stocks

J6/JFH-1 virus genotype 2a (provided by C.M. Rice, Rockefeller University) was prepared as described [22]. Virus infected cells were grown at 37°C with 5% carbon dioxide for 16 days when supernatants were cleared by centrifugation and filtered through a 0.45 μm membrane. To ensure media was free of Phenol Red (an estrogen mimic), supernatants were dialyzed twice over 48 hr, using 1X PBS and Slide-A-lyzer Dialysis Cassettes G2 (cutoff of 10,000 MW; Pierce). Virus stocks were titrated in Huh7.5 cells, as described below.

### HCV titer determination

Viral titers were determined by focus forming assays as previously described by Silberstein et al. 2010 and [23] with some exceptions: The cells were fixed at 2 dpi, fixed with 2% formaldehyde and stained with HCV anti-core antibody and developed using an Avidin-HRP conjugated with Diaminiobenzidine (DAB) as substrate (Vectastain ABC Kit, Mouse IgG Vector Laboratories). Stained foci were counted in quintuplicate wells and data represent the absolute mean foci number.

### Virus Inhibition assays

Huh7.5 cells (seeded at 5x10^4^ cells/100ul/well density) were pre-treated for 24 hr with E2, Tam, G1, and/or MMP inhibitors. Media was removed and cells were infected with J6/JFH1 for 6 hr, the supernatant was removed and treatment continued for 2 additional days (Table 1A). Cells were pre-incubated with G15 for 1 hour, and then incubated with E2 or G1 before and after HCV infection (Table 1B).

**Table 1:** Experimental procedure for evaluating virus growth by FFA.

	Treatment	Day 0 Cell Seeding	Day 1 Pre-Treatment	Day 2 Infection and Treatment	Day 4 Endpoint
**A**	Virus growth with E2, G1, Tam +/- MMP inhibitors.	5x10^4^ Huh7.5 cells in 96 well plate.	E2, G1 or Tam for 24 hr in the presence or absence of an MMP inhibitor.	Media removal and virus inoculation. After 6 hr, media was replaced by same treatment as on Day 1 for 48 hr.	Media removal and cell fixation, immuno-histochemistry for foci staining for foci quantitation.
**B**	Virus growth with G15 pre-incubation.	5x104 Huh7.5 cells in 96 well plate.	Pre-incubation with G15 for 60 min. Then, addition of E2 or G1 for 24 hr in the presence of G15.	Media removal and virus inoculation. After 6 hr, media was replaced by same treatment as on Day 1 for 48 hr.	Media removal and cell fixation, immuno-histochemistry for foci staining for foci quantitation.

(A) Protocol for E2, G1 or Tam treatment of HCV-infected cells in the presence or absence of MMP inhibitors. Results are shown in Figs 1D, 2A, 2B and 5A–5E. (B) Protocol for GPR30 pathway confirmation through pre-incubation with the specific-GPR30 antagonist G15. Results are shown in Fig 2C and 2D.

**Fig 1:**
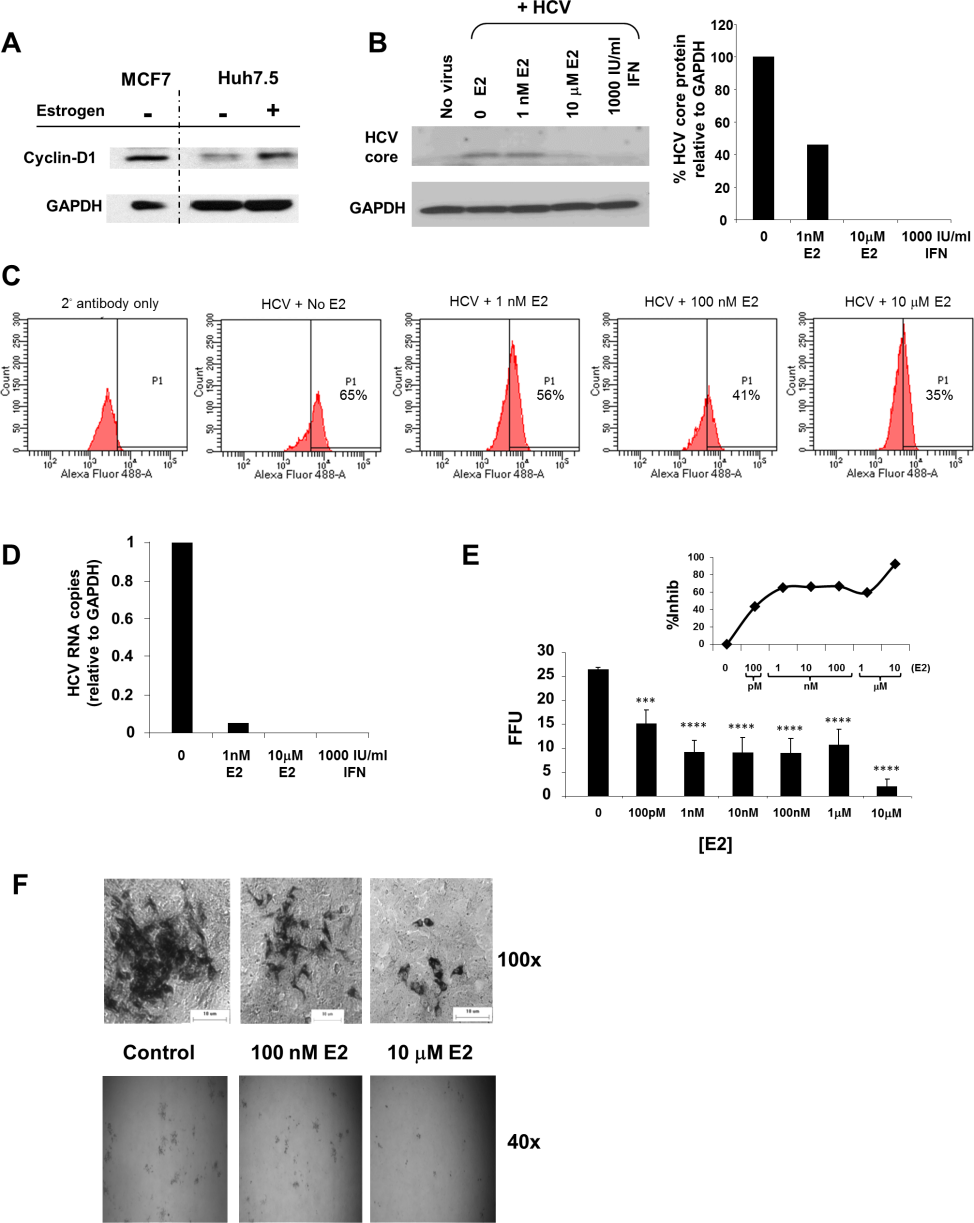
Estrogen abrogates HCV infection. (A) Huh7.5 cells respond to estrogen treatment measured by cyclin-D1 expression. (B) HCV core protein in J6/JFH1-infected Huh7.5 cells treated with E2 or Universal Type I IFN. (C) HCV intracellular core protein in J6/JFH1-infected Huh7.5 cells treated with E2, as measured by Flow Cytometry. (D) HCV RNA quantitated relative to GAPDH RNA levels from infected Huh7.5 cells treated with E2 or IFN. (E) Virus growth measured by absolute mean focus forming units (FFU). Error bars indicate the statistical standard deviation from the mean (±SD). Statistical significance is indicated by asterisks where: (*** = P ≤ 0.001; **** = P ≤ 0.0001). (F) Representative images of foci after 48 hr of E2 treatment. Magnification 40X (*below*); 100X (*above*).

**Fig 2:**
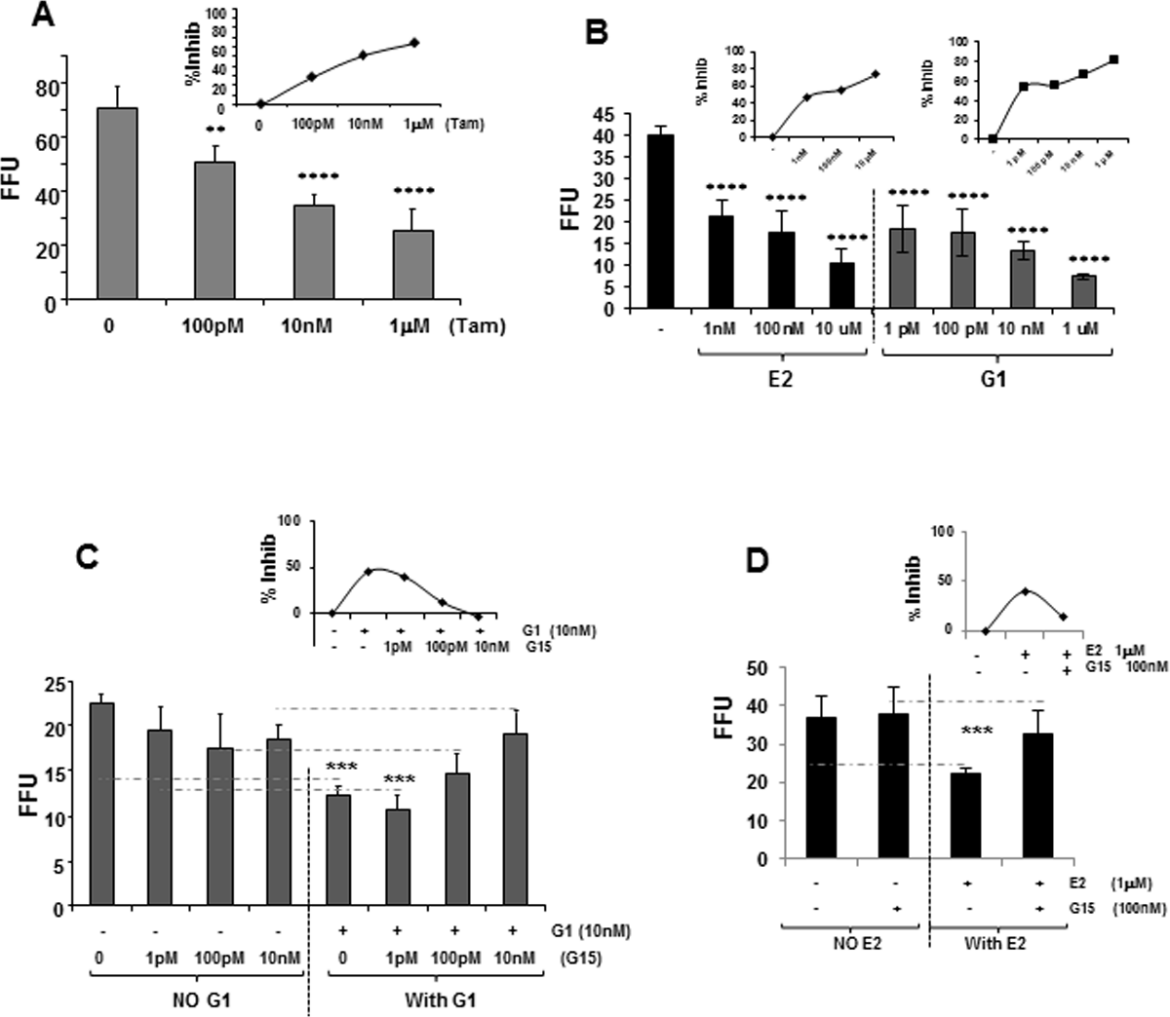
GPR30 signaling inhibits HCV growth. (A) Increasing levels of Tamoxifen, (B) E2 and G1 inhibit HCV. (C) Pre-incubation with GPR30 specific antagonist, G15, reverses the inhibitory effect of G1 and (D) E2 on hepatoma cells.

In all inhibition experiments, viral growth was determined by focus forming assay. Stained foci were counted in triplicate or quintuplicate wells and data represent the absolute mean foci number or percentage of inhibition [% Inhib = (C-T) x 100/C] (where C is the mean foci number of the control group (solvent or with antagonist/inhibitor alone) and T is the mean foci number of the treated group.

### HCV RNA quantification

Total cellular RNA was extracted from Huh7.5-infected cells using RNeasy minikit, following the manufacturer’s recommendation (Qiagen), and quantitated using a Nanodrop 1000 Spectrophotometer (Thermo Fisher Scientific, Inc.). RNA was analyzed by quantitative RT-PCR as described [20].

### Effect of E2, G1 and G15 on HCV receptors expression

**Fig 3:**
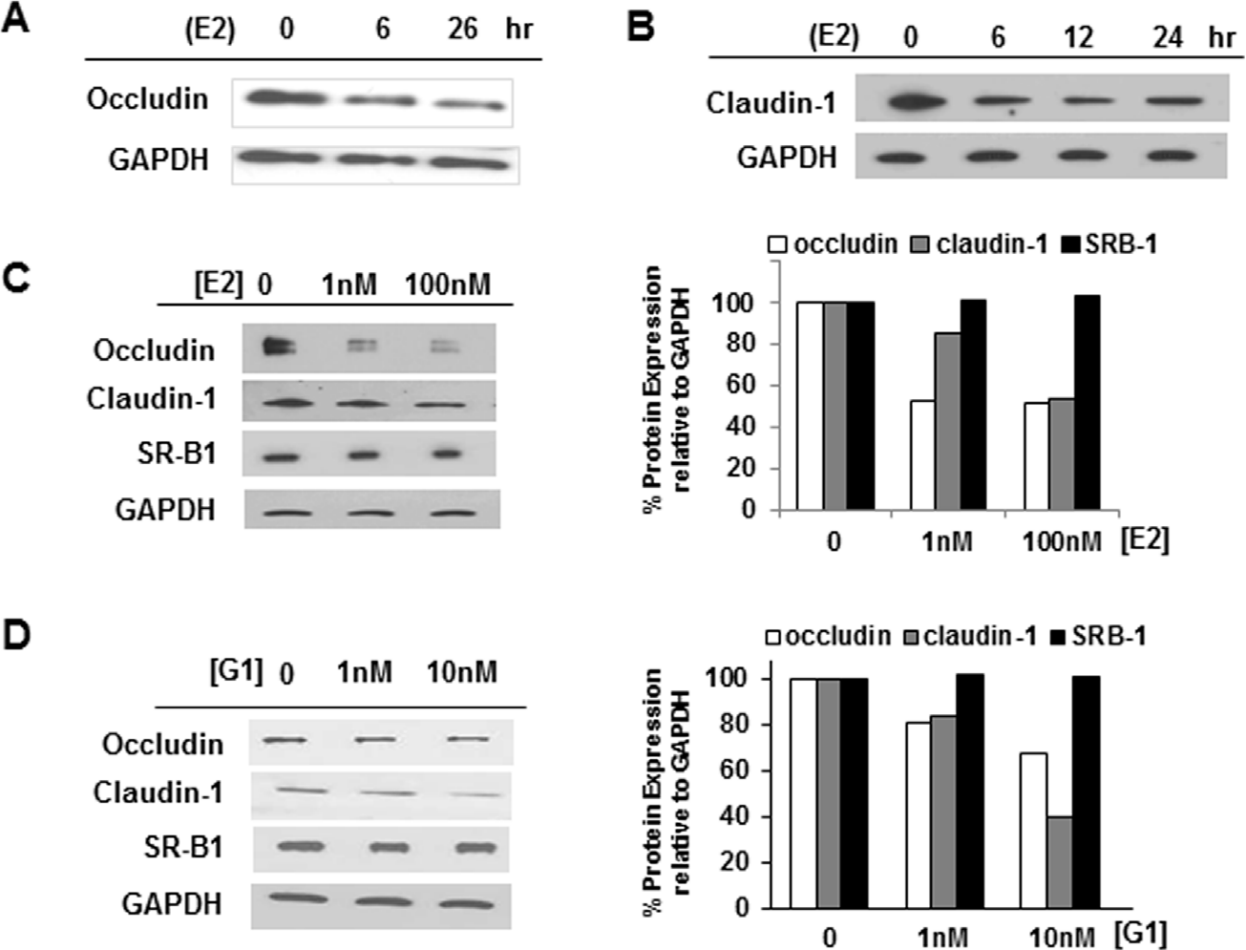
Effect of E2 on occludin and claudin-1. (A) Immunoblots of occludin and (B) claudin-1 protein from Huh7.5 cells treated with 10 μM E2. (C) Expression of HCV receptors occludin, claudin-1 and SR-B1 in Huh7.5 cells treated with increasing amounts of E2 or (D) G1. Intensity of immunoblot bands was quantified relative to GAPDH protein levels (*right*).

Huh7.5 (2.5x10^6^) cells were seeded in T25 flasks in 10% Charcoal Stripped FBS Phenol Red Free media (CSFBS DMEM-E2 minus media) for 24 hr. Next, media was replaced by 0.5% CSFBS DMEM-E2 minus media. After 24 hr of starvation, cells were treated with 10 μM β-estradiol (in 0.5% CSFBS DMEM-E2 minus) for 0, 6, 12 and 24hr. Alternatively, cells were treated with β-estradiol (1 nM and 100 nM), or with GPR30 agonist G1 (1 nM and 10 nM), in 0.5% CSFBS DMEM-E2 minus, for 24 hr (Fig 3).

**Fig 4:**
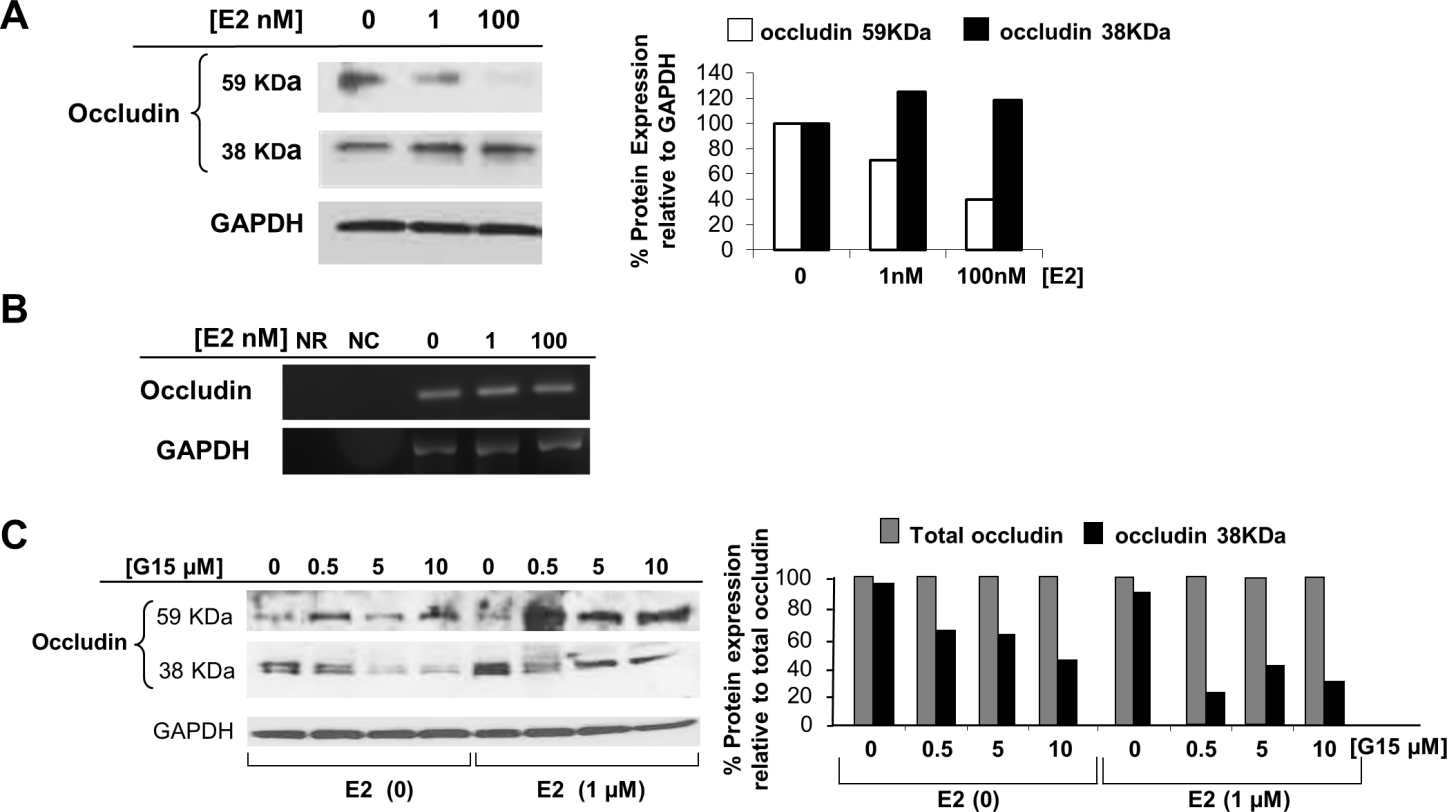
E2 treatment leads to occludin cleavage in Huh7.5 cells. (A) Detection of full-length (59 KDa) and truncated (38 KDa) occludin in Huh7.5 cells after treatment with increasing amounts of E2 for 24 hr. (B) Occludin and GAPDH cDNA in Huh7.5 cells following treatment with increasing levels of E2 for 24 hr. NR, RNA/no cDNA control; NC, no RNA/no cDNA control. (C) Immunoblot of full-length and truncated occludin in G15- and E2-treated cells. Intensity of immunoblot bands was quantified by imaging analysis and the percentage of total occludin was calculated relative to GAPDH protein levels. The percentage of truncated (38 KDa) occludin was determined as relative to corrected total occludin, *right*.

Inhibition of E2 effects on occludin expression was studied using Huh7.5 cells pre-incubated with increasing concentrations of G15 (0, 0.5, 5 and 10 μM) for 1 hr, and then 1 μM E2 was added for additional 20 hr. It should be noted that G15 was present in the media throughout the whole incubation period (Fig 4).

### Immunoblot analysis

Immunoblots were performed as previously described [20]. The SuperSignal West Pico or Femto Chemiluminescence Substrate kits (Pierce) were used for detection as recommended by the manufacturer. Intensity of immunoblot bands was quantified by imaging analysis in a SyngeneG:box and the percentage of occludin forms were calculated relative to GAPDH protein levels.

### Extraction and quantification of occludin mRNA

Total RNA was extracted from Huh7.5 cell lysate using the Qiagen QIAamp Viral RNA mini Kit following the manufacturer’s protocols and reverse-transcription was performed using the Invitrogen SuperScript III cDNA synthesis kit. The resulting cDNA was then amplified using the oligonucleotides for occludin as described in [24] and for GAPDH previously described in [18].

### Subcellular protein fractionation

Huh7.5 (10^7^) cells were seeded in T25 flasks containing DMEM-E2 minus for 24 hr. Subsequently, cells were washed twice with DMEM-E2 minus (serum free) and treated with E2, in serum free media, for different time points. Total cells were harvested, counted (Vi-Cell XR, Beckman Coulter) and membranes were separated from the cytosol and nucleus using the Subcellular Protein Fractionation Kit for cultured cells (Thermo Scientific). Culture supernatants were cleared by centrifugation and concentrated using Amicon Ultra Centrifuge Filters 30K (Millipore) and 50 μg of total membrane proteins and the equivalent volume to 10^5^ cells from a culture supernatant were analyzed by immunoblot.

### Flow cytometry analyses: Detection of occludin and HCV core proteins

Cells were collected by incubation with Accumax™ Cell Detachment Solution (Sigma), pelleted (300xg for 5 minutes), washed twice with Flow cytometry staining Buffer (R&D Systems) and blocked with Human Fc Blocking Solution (R&D Systems) for 15 minutes at 4°C. Cell viability was established with the LIVE/DEAD Fixable Aqua Dead cell stain kit (Life Technologies), following manufacturer instructions.

For HCV core staining, fixation was carried out by using Flow cytometry Fixation Buffer (R&D Systems) and incubating the cells for 10 minutes at room temperature. Permeabilization was performed with Flow cytometry permeabilization Buffer (R&D Systems). After an incubation of 15 minutes at room temperature, cells were stained with 1 μg (per 10^6^ cells) of mouse anti-HCV core monoclonal antibody (Pierce, Thermo Scientific) and Alexa Fluor 488-conjugated goat anti-mouse IgG (H+L) (Life Technologies) diluted 1/2000. All flow cytometry studies were performed on a FACS Calibur cytometer (BD Biosciences, San Diego, CA, USA) with data analyses conducted using FlowJo 7.6.3 software (Tree Star, Inc., Ashland, OR, USA).

### MMP-9 knock down with siRNA

Huh7.5 (10^6^) cells were seeded and reverse transfected in 6 well plates, in DMEM-E2 minus for 24 hr, with 20 nM final concentration of small interfering RNA (siRNA) following the manufacturer protocols (Dharmacon, Inc., Lafayette, CO). siRNA, Dharmafect 4 Transfection reagent, 5X siRNA buffer and siGLO Transfection Indicator were obtained from Dharmacon. siRNA specific to MMP-9 (ON-TARGET plus Human MMP-9 siRNA “SMART pool”, Target sequences: GCAUAAGGACGACGUGAAU, GGACCAAGGAUACAGUUUG, GCGCUCAUGUACCCUAUGU, and GAACCAAUCUCACCGACAG). MMP-9 sequence-Scrambled siRNA was used as a negative control (ON-TARGET plus Non-Targeting siRNA, Target sequence: UGGUUUACAUGUUUUCCUA).

### Statistical analysis

All experiments were repeated a minimum of three times. Data were expressed as mean ± standard deviation. Differences between treatment and control groups were determined by One-way analysis of variance (ANOVA) followed by Bonferroni posttest. Significance was assumed to be at two tailed p<0.01 (* = P≤ 0.05; ** = P ≤ 0.01; *** = P ≤ 0.001; **** = P ≤ 0.0001).

## Results

### β-estradiol down-regulates HCV

To study the effects of E2 on HCV growth, we first determined that Huh7.5 cells were responsive to E2 treatment. ER activation induces transcription of cyclin-D1 [25]. We treated Huh7.5 cells with 100 nM E2 for 48 hr and they responded to E2 treatment as demonstrated by increased cyclin-D1 expression (Fig 1A); MCF7 cells were used as a positive control for the cyclin-D1 antibody. Ethanol, the E2 solvent, was used in all experimental controls. To determine the effect of E2 on HCV growth, cells were pretreated with E2 at doses ranging from physiological (1 nM) to HRT levels (10 μM). After 24 hr, cells were infected with J6/JFH-1 [22], and treated for an additional 72 hr with E2 or IFNα (Fig 1 and Table 1A). To ensure that our system would respond as previously reported, HCV infection was monitored by expression of core protein, HCV RNA and viral growth. HCV core protein expression decreased by 60% with 1 nM E2 treatment and by 100% with 10 μM E2 treatment or IFNα as detected by immunoblot (Fig 1B). When analyzed by flow cytometry, there was a dosage-dependent decrease in HCV core protein with increasing concentrations of E2 (Fig 1C). Down-regulation of HCV protein was consistent with a decrease in relative HCV RNA levels, measured by quantitative reverse-transcriptase real-time PCR (TaqMan; Fig 1D). When treated with 1 nM E2, HCV RNA levels decreased to only 5% of the untreated control samples (0). In addition, virus growth decreased in a dosage-dependent manner, measured in focus forming units (FFU). Increasing concentrations of E2 (100 pM-10 μM) resulted in decreased HCV foci number, with virus inhibition of 43–92% (Fig 1E and 1E inset). Interestingly, E2 treatment reduced foci number and also resulted in decreased size of the foci (Fig 1F), suggesting that E2 also affects a viral mechanism post-initial infection, such as replication or viral spread.

### Estrogen mediates control of HCV through the GPR30 pathway

E2 signaling is promoted by ER-α, ER-β or GPR30 [8]. In some cells, Tam inhibits E2 by competitive binding to the ER(s) [26, 27, 28]; however, both E2 and Tam activate GPR30. Increasing amounts of Tam added 24 hr before infection and for 48 hr after infection inhibited HCV growth (Table 1A and Fig 2A). Viral inhibition (30–60%) was observed with dose increases in Tam (100 pM-1 μM), similar to the results observed for E2 treatment (Fig 1E) and consistent with activation of GPR30 by both E2 and Tam. These data are consistent with a previous report, whereby Tam inhibited HCV growth [12].

Signaling through GPR30 was confirmed with a GPR30-specific agonist known as G1 [28]. Increasing concentrations of E2 (1 nM-10 μM) or G1 (1 pM-1 μM) decreased HCV foci number, with virus inhibition of 50–70% for E2 and 55–80% for G1 treatment (Fig 2B). Inhibition of HCV by E2 and G1 (Fig 2B, insets) display the same shaped curve, indicative of a high-potency receptor agonist that exhibits similar behaviors with respect to inhibition of HCV growth. These data suggest that E2 and G1 inhibited HCV in a dosage-dependent manner through the GPR30 receptor.

To further confirm the involvement of GPR30, cells were pre-incubated for 1hr with increasing concentrations of G15, a GPR30-specific antagonist [29]. G1 or E2 were added to the cells before and after HCV infection, in the presence of G15. Treatment with G15 alone had no significant effect on HCV growth (Fig 2C and 2D, left), but increasing concentrations of G15 reversed (or blocked) the inhibitory effect of G1 (Fig 2C, right) and E2 (Fig 2D) on HCV growth. To determine the statistical significance of the effect of G15 on G1 action, we compared the treatments by pairs (Fig 2C. dashed lines). HCV growth was inhibited significantly with 10 nM G1 and in combination with low levels of G15 treatment (0 and 1 pM G15). At 100 pM and 10 nM G15, HCV growth was restored in the presence of G1. There was no significant difference between the cells that received G15 alone and those that received G15 and E2, confirming that G15 reversed the inhibitory effect of E2 (Fig 2D). Taken together, these data demonstrate that HCV was inhibited by E2, Tam and G1 and the inhibition of HCV was blocked by the addition of G15, a specific antagonist of the GPR30 receptor, indicating that E2 antagonizes HCV via the GPR30 pathway. These data do not exclude the possibility that the nuclear ERs are also acting on HCV.

### Mechanism of action: Effect of E2 on occludin and claudin-1

In cervical vaginal cells, E2 led to the cleavage of occludin into a truncated-inactive form through activation of host proteinases [17]. To elucidate the mechanism of E2 action on HCV growth, we observed the expression of the HCV receptors/tight junction proteins occludin and claudin-1. After E2 treatment, a time-dependent decrease was observed for both occludin and claudin-1 protein at HRT levels (Fig 3A and 3B). At physiological levels, occludin and claudin-1 expression was down-regulated by E2 as well, but not the scavenger receptor-B1 (SR-B1; Fig 3C), which is also an HCV receptor. Increasing amounts of G1 caused a similar pattern of expression, indicating that GPR30 activation in liver cells resulted in a specific decrease in occludin and claudin-1 expression (Fig 3D).

Huh7.5 cells treated with increasing amounts of E2 yielded a dose-dependent decrease in full-length 59 KDa occludin with a concomitant increase in a 38 KDa form of occludin (Fig 4A). A highly-sensitive chemiluminescence substrate (FEMTO; Pierce) was required to visualize the truncated form of occludin. No effect of E2 was observed when occludin cDNA levels were visualized by RT-PCR amplification (Fig 4B). To confirm that GPR30 signaling resulted in proteolytic cleavage of occludin protein, Huh7.5 cells were pre-treated with G15 (Fig 4C). As expected, the truncated form of occludin increases with E2 treatment and no G15 (0 G15; Fig 4C). As the level of G15 was increased, the relative amount of 38 KDa occludin also decreased (Fig 4C, *right*), indicating that occludin cleavage, was blocked by G15. Note that the truncated, 38 KDa, form of occludin is present in the absence of E2, indicating that there is some constitutive cleavage of occludin that can be blocked with G15 alone, due to inhibition of GPR30. Because G15 is specific for the inhibition of GPR30, these data support the role of E2 in GPR30 activation leading to occludin cleavage from a 59 KDa form to a 38 KDa form.

### Modulation of HCV by Matrix Metalloproteinase

**Fig 5:**
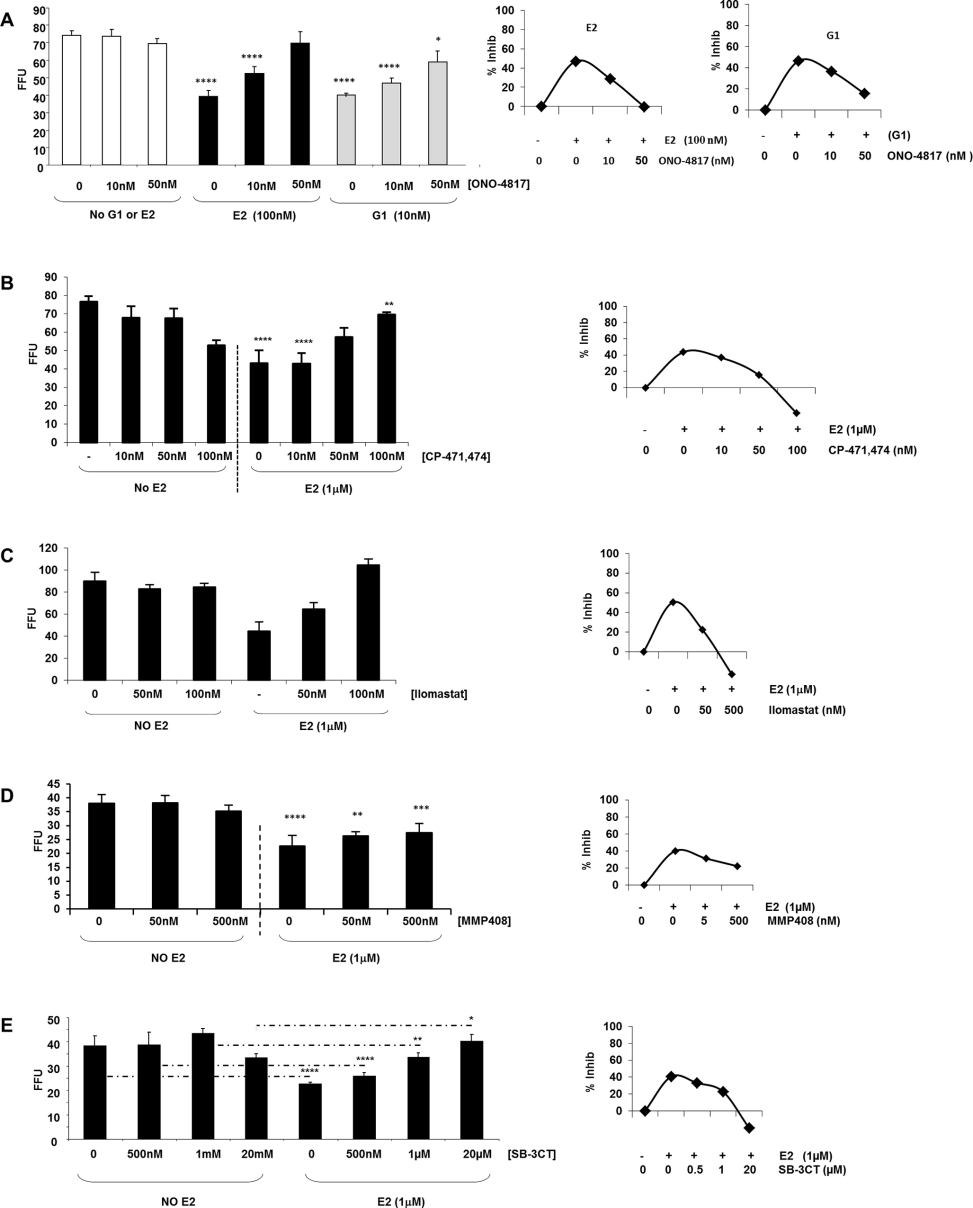
E2 modulates matrix metalloproteinase to inhibit HCV growth. (*A*) Response of increasing concentrations of the broad-spectrum MMP inhibitor, ONO-4817 on J6/JFH1-infected Huh7.5 cells, in the presence or absence of E2 (*center panel*), and G1 (*right panel*) on HCV growth. Increasing concentrations of the broad spectrum MMP inhibitors (*B*) CP471,474 (C) Ilomastat (D) MMP408 or (E) SB-3CT. Data represent the absolute mean foci number of three independent experiments and error bars represent the standard deviation from the mean (±SD). Statistical significance is expressed as asterisks where; (* = P≤ 0.05; ** = P ≤ 0.01; **** = P ≤ 0.0001).

Previously, E2 was shown to lead to occludin cleavage by MMP-7 in cervical-vaginal cells [17, 18]. HCV-infected Huh7.5 cells were treated with E2 or G1 in the presence of an MMP inhibitor (Fig 5A–5E). HCV growth was measured by focus forming assay with increasing concentrations of the broad range inhibitor ONO-4817 [30]. As expected, this MMP inhibitor exhibited no effect on HCV growth when used alone (Fig 5A, *left*). However, when 50 nM of the MMP inhibitor was used in combination with E2 or G1, the growth of HCV was restored (Fig 5A, *center* and *right* with insets), suggesting that it reversed E2’s inhibition of HCV. Statistical calculations were made between the corresponding controls in the far left panel (no G1 or E2) and the addition of hormone/agonist in both right panels of Fig 5A. E2 (100 nM) and G1 (10 nM) resulted in decreased HCV foci number, with virus inhibition around 50% (Fig 5A and 5A inset). In the presence of the MMP inhibitor, ONO-4817, the percent inhibition decreased, meaning that the MMP inhibitor blocked or reversed the effects of E2 and G1 on HCV growth. Complete restoration of virus growth in the presence of hormone or GPR30 agonist and MMP inhibitor is demonstrated as non-significant because the results are similar to the respective controls. The restoration of virus growth in the presence of hormone and a broad spectrum MMP inhibitor suggest that one or more MMPs are responsible for the inhibition of HCV in response to E2 activation of GPR30 (summarized in Table 2).

**Table 2:** Identification of the MMP responsible for HCV growth inhibition.

Inhibitor	Target MMPs	Reversed E2 Inhibition of HCV
**ONO-4817**	**2, 3, 8, 9, 12, 13**	**+**
**CP-471,474**	**1, 2, 3, 9, 13**	**+**
**Ilomastat**	**1, 2, 3, 8, 9**	**+**
**(GM 6001)**	** **	** **
**MMP408**	**3, 12, 13**	**-**
**SB-3CT (μM)**	**9**	**+**
**SB-3CT (nM)**	**2**	**-**
**DMSO (vehicle)**	**-**	**-**

MMP inhibitors target different groups of MMPs [30, 31, 32, 33, 34]. MMP Inhibitors ONO-4817, CP-471,474, Ilomastat, and SB-3CT reversed the effect of E2 on HCV growth (Fig 5A–5C and 5E, Table 2) while MMP408 did not (Fig 5D). ONO-4817 reversed E2’s effects, indicating that MMP-2, -3, -8, -9, -12 or -13 were potentially involved in HCV down-regulation (Table 1). CP-471,474 activity ruled out MMP-8 and -12 (Fig 5B) while ONO-4817 activity also ruled out MMP-1 (Table 1). Ilomastat can inhibit MMP-1, -2–3, -8, and -9 and was effective at reversing E2’s inhibition of HCV, indicating that MMP-2, -3 and -9 were possible candidates (Table 2). However, not all MMP inhibitors reversed the effect of E2; MMP408 inactivity ruled out MMP-3, -12 and 13 (Fig 5D), leaving only MMP-2 and MMP-9 as possible options (Table 1). While SB-3CT can inhibit MMP-2 with a Ki = 14nM, a higher concentration is needed to inhibit MMP-9, Ki = 600nM [32]. SB-3CT was not effective at reversing the effect of E2 at nanomolar levels but was effective at micromolar levels, narrowing the possible candidates down to MMP-9 (Fig 5E, Table 2). Consistent with our findings, Razandi et al. found that E2 signals through GPR30, leading to activation of MMP-2 and MMP-9 in breast cancer cells [35].

### MMP-9 modulates occludin cleavage

**Fig 6:**
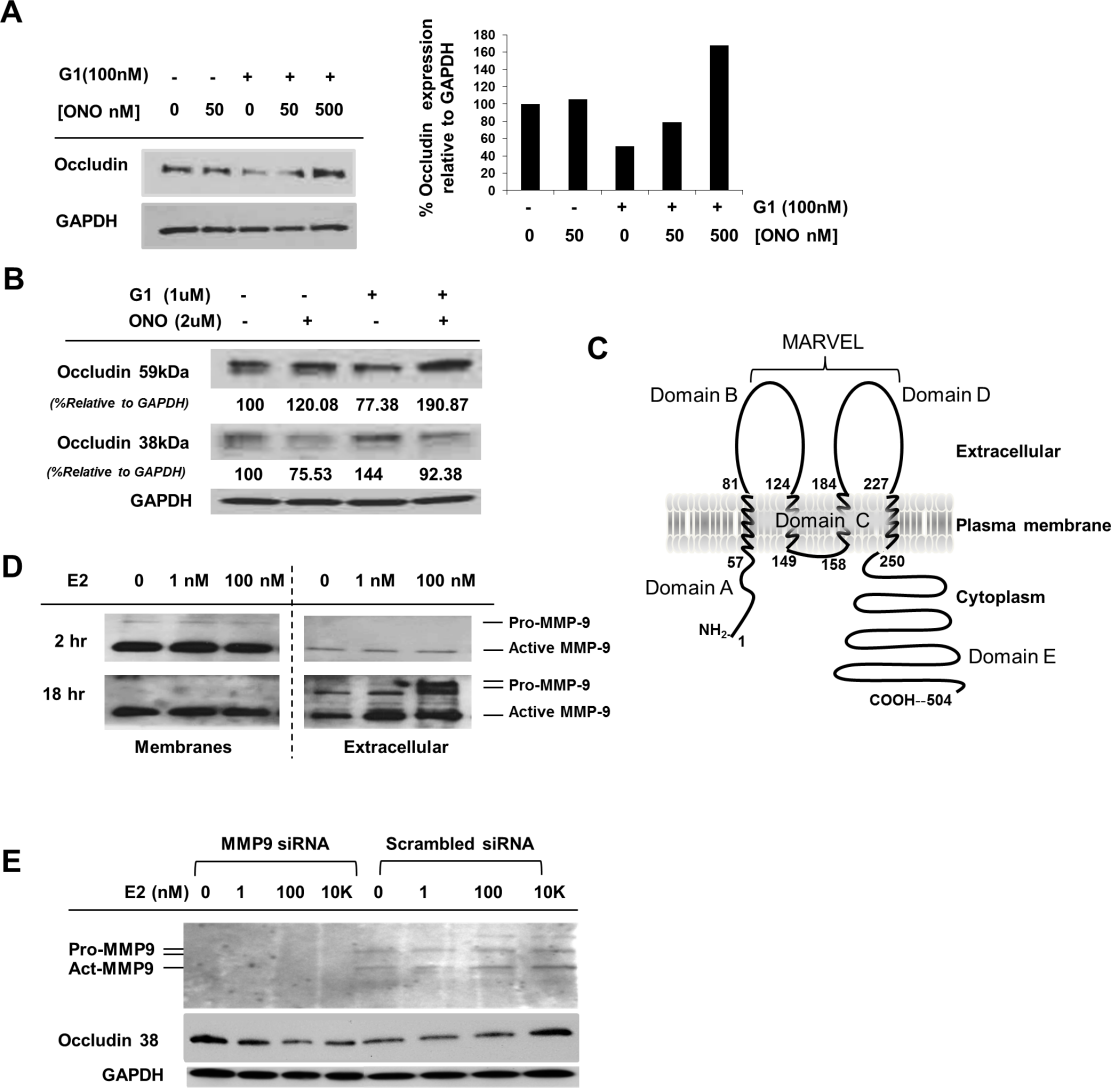
MMP-9 activation mediated by E2 modulates occludin. (A) Immunoblot of occludin down-regulation by G1, reversed by ONO-4817 (ONO). Protein expression relative to GAPDH was quantitated in a SyngeneG:box (*right*). (B) Immunoblot of full-length and truncated forms of occludin in Huh7.5 cells treated with G1 and ONO-4817 and the percentage of occludin forms were calculated relative to GAPDH protein levels (values below each band). (C) Schematic representation of occludin (504 amino acids). (D) Immunoblot of pro-MMP-9 (92 KDa) and active MMP-9 (~64 KDa) in Huh7.5 cell membranes and conditioned media (extracellular) following E2 treatment. (E) Immunoblot of MMP-9 in supernatant (extracellular) after siRNA transfection and E2 treatment (0, 1, 100, 10,000 nM). Cleaved occludin (38 KDa) is shown and GAPDH is shown as a gel loading control.

To link GPR30 activation by E2 with MMP-9 cleavage of occludin, Huh7.5 cells were treated with G1 in the presence or absence of increasing concentrations of the MMP inhibitor, ONO-4817. Consistent with our data shown in Fig 3D, full-length occludin levels decreased with G1 treatment alone (+), which was reversed with increased amounts of ONO-4817 (Fig 6A), suggesting that G1 leads to cleavage of occludin by an MMP. As seen before with E2 treatment (Fig 4A), two forms of occludin appeared with G1 treatment, a 59 KDa and a 38 KDa form (Fig 6B). The truncated form of occludin was present without any drug; decreased with the addition of MMP inhibitor and increased with G1 treatment alone. The 59 KDa band increased concomitantly with a decrease in the 38 KDa band in the presence of ONO-4817, consistent with the integrity of the full-length protein due to inhibition of an MMP. The 38 KDa form was observed in the absence of G1, suggesting that an MMP is constitutively active and consistent with an increase in the 59 KDa form in the presence of ONO-4817 alone (Fig 6B).

We observed both full-length and truncated forms of occludin on immunoblots that were probed with an antibody directed at its C-terminus. Because 38 KDa corresponds to approximately 345 amino acids (~110 Daltons/aa) containing the C-terminus, the MMP-9 proteinase digestion site must lay between domains C and D, somewhere between the plasma membrane and the extracellular side of occludin (Fig 6C).

To identify the cellular location of active MMP-9, sub-cellular protein fractionation was performed after E2 treatment and the pro- (92 KDa) and active- (~64 KDa) forms of MMP-9 [36, 37] were detected by immunoblot in the membrane fraction and in the supernatant as well (Fig 6D). Membranes were extracted and the conditioned media (extracellular fraction) was concentrated. Both pro-MMP-9 and active MMP-9 were up-regulated by E2 in the supernatant after 18 hr of E2 treatment. No change was observed in active, membrane-associated MMP-9, but an increase in the extracellular fraction for both forms of MMP-9 was observed with E2 treatment. This suggests that proteolytic cleavage did not occur in Domain C but occurred in the extracellular Domain D, where active MMP-9 was secreted upon E2 treatment (Fig 6C and 6D). When MMP-9 expression was knocked-down with siRNA, both pro- and active forms were not detectable in the supernatant (Fig 6E). Treatment with increasing levels of E2 did not result in an increase in the cleaved form of occludin when MMP-9 was absent (MMP9), but only when MMP-9 was present (scrambled siRNA; Fig 6E). These data are consistent with a role for MMP-9 in the cleavage of occludin.

## Discussion

**Fig 7:**
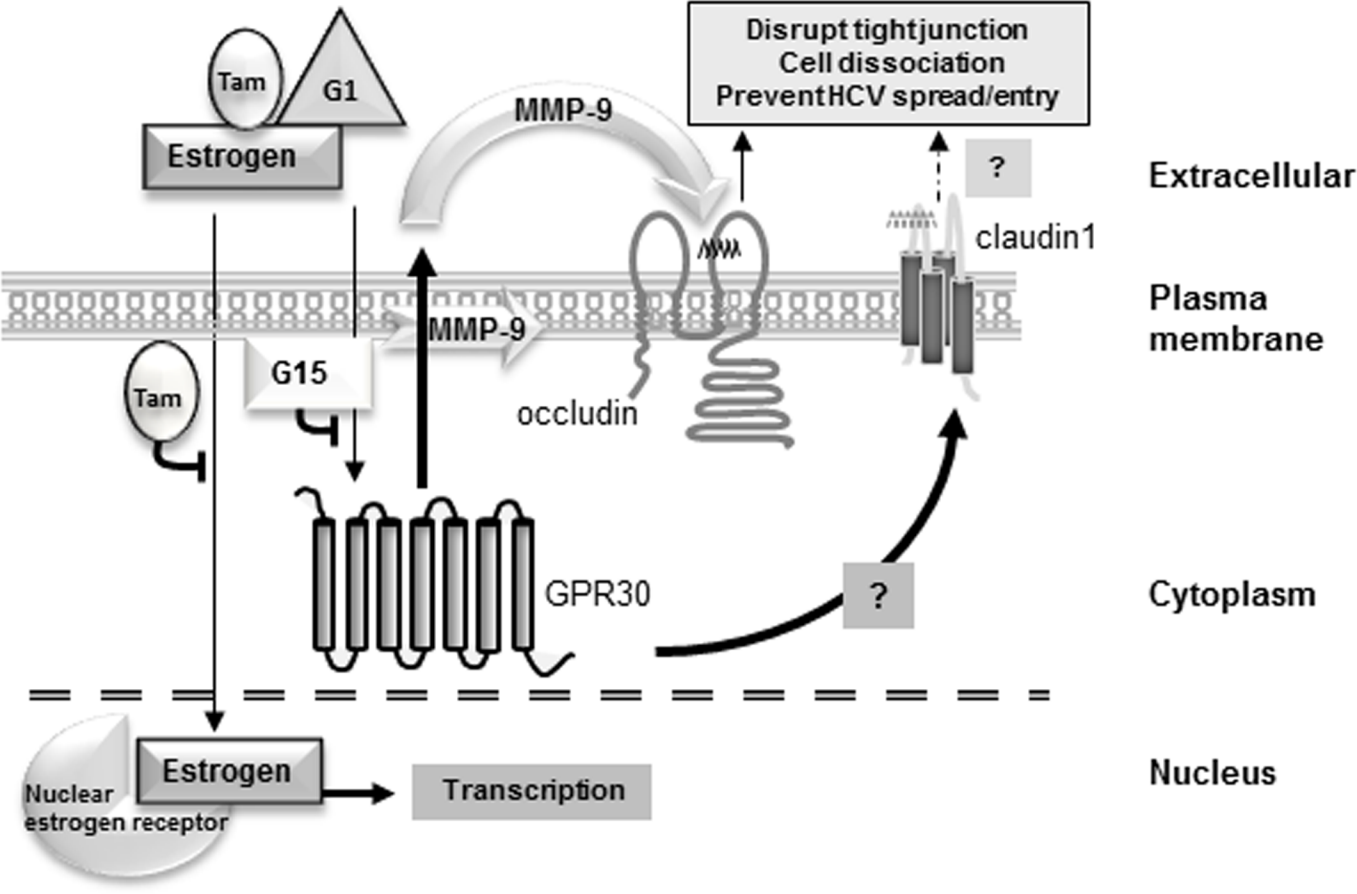
Schematic of the E2 signaling pathway controlling HCV entry and spread in liver cells. The nuclear receptor mediated pathway is activated by E2 and blocked by Tamoxifen (Tam) in most cell types. Activation of GPR30 is inhibited by G15 and initiated by E2, Tam or G1, leading to activation/secretion of MMP-9, which cleaves occludin in extracellular Domain D, resulting in a defective HCV receptor.

Here, we present the molecular pathway by which E2 causes inhibition of HCV spread and/or entry through down-regulation of functional occludin (Fig 7). Both Tam and E2 inhibited virus growth (Figs 1 and 2), but to identify the ER used to down-regulate HCV, a GPR30-specific-agonist (G1) or a GPR30-specific-antagonist (G15) were employed. G15 was capable of reversing G1 and E2 effects (Fig 2) leading to the conclusion that HCV inhibition occurred through activation of the GPR30 receptor. The effect of E2 on HCV growth and occludin cleavage was mimicked by G1 and reversed by G15, confirming the involvement of GPR30 (Figs 2–4). The field of candidate MMPs responsible for limiting HCV growth was narrowed to MMP-9 with the use of broad and narrow range MMP inhibitors (Fig 5, Table 2). G1, which is a specific activator of GPR30, caused an increase in the truncated form of occludin, which was reversed with MMP inhibitors or by an MMP-9-specific siRNA (Fig 6); confirming that the negative effects of GPR30 activation can be blocked by loss of MMP-9 function and consistent with MMP-9 activation leading to occludin cleavage. These findings were reported in breast cancer cells by Razandi et al. (2003)[35].

We found that E2 activated GPR30, increasing MMP-9 activation and export to the extracellular space leading to cleavage of occludin in Domain D (Figs 6 and 7).

An intact Marvel Domain D in occludin (Fig 6C) is required for HCV entry [15, 24, 38]. This second extracellular loop (Domain D) forms the tight junction/cell barrier between cells [39] and cleavage of occludin at this second extracellular loop disrupts occludin-occludin and occludin-claudin-1 interactions [18]. These data are consistent with the observation that HCV cell-to-cell spread is inhibited after occludin cleavage (Fig 1E). Because Huh7.5 cells are not capable of polarization [40, 41, 42], we could not determine if the TJ integrity was affected by E2-induced cleavage of occludin, however this line of study should be pursued in the future.

Because pro-MMP-9 and active MMP-9 appeared in the extracellular fraction (Fig 6D) and a basal level of occludin cleavage (Fig 4A) was present in the absence of E2, we speculate that other factors might be involved in the up-regulation of MMP activity. This is consistent with previous studies showing up-regulated MMP-9 mRNA expression in HCV patients [43] and in virus-infected Huh7.5.1 cells [44]. While little is known about the activation of the MMPs, TIMPs (tissue inhibitor of the MMPs) are physiological, natural MMP inhibitors that regulate MMP activity by complexing with unprocessed forms of MMPs [45]. We cannot exclude the possibility that E2 or HCV or both, may be playing a role in pro-MMP-TIMP associations or dissociations. TIMPs might limit cleavage of occludin to a basal level, consistent with the appearance of the 38 KDa occludin in the absence of E2 or G1 (Figs 4A and 6B). This basal level may be altered in the presence of E2, leading to MMP up-regulation, although the mechanism is not clear and TIMP activities are not well understood.

Among normal MMP functions are the destabilization of tight junctions, degradation of extracellular matrix proteins, involvement in cell motility, wound repair and tissue invasiveness. The normal healing process after liver injury involves the accumulation and deposition of collagen (scar formation) and matrix reorganization (scar remodeling) and is a dynamic process whereby MMPs and TIMPs are involved. Excess healing results in liver fibrosis, which can lead to cirrhosis over time. Voloshenyuk and Gardner reported that E2 reduces the distribution of collagen in the hearts of rats and affects the equilibrium between TIMP1-MMP-9 and TIMP2-MMP-2 and MMP secretion [46]. Considering that one of the targets of MMP-9 is collagen, our data may suggest an explanation for the lower levels of fibrosis in pre-menopausal women compared with post-menopausal women and men that are infected with HCV. More studies need to be done to establish the role of MMP-9 and E2 in modulation of collagen accumulation and the prevention of fibrosis.

In contrast to our results, it has been reported previously that E2 inhibits HCV exclusively through ERα because G1 had no effect on HCV virion production [47]. However, in that study, cells were treated with E2 after and during infection, and only 2 hr before HCV infection. We found that E2 down-regulated HCV through GPR30 when cells were pretreated for 24 hr and treated after infection (48 hr); (Table 1A, Fig 2). As seen in Fig 3A and 3C, 6 hr of treatment (at 10 μM E2) and 24 hr of treatment (at 1 and 100 nM E2) were required for a decrease in occludin protein, thus, explaining differences that the two labs observed. Furthermore, Murakami and coworkers treated HCV for 5 days, comparing E2 with Tam and other SERMs [12]. They saw no inhibitory effect of E2 or G1 on HCV, while the other SERMs inhibited the virus. Therefore, they concluded that GPR30 was not involved. Because E2 has a short half-life (13–17 hr) and Tam has a half-life of 5–7 days, no effect would be seen by E2 if only looking at a 5 day time point. In fact, we observed a rescue of virus after 48 hr of E2 treatment (data not shown), indicating that E2 activity had been extinguished and explaining the negative effect over 5 days of treatment. Our work, however, does not exclude the possibility that ER-α may also play a role in HCV control.

Our demonstration that E2 down-regulates HCV entry and spread through the GPR30 pathway by promoting occludin cleavage by MMP-9 represents a new understanding of a cellular pathway that participates in an innate antiviral response. Huet et al. (2011) also found that occludin was cleaved by MMP-9, but in human corneal epithelial cells [48]. These data set the stage for further investigations into novel therapies that target host innate defenses, and underscores the importance of employing HRT in post-menopausal women infected with HCV. Our work shows that E2 provides a less than ideal environment for the virus to spread by reducing the availability of HCV receptors. Activation of the GPR30 pathway by E2 explains the sexual dichotomy that occurs during HCV infection and why premenopausal women suffer less-severe pathologies and postmenopausal women progress towards disease as poorly as men.
